# Identification of a Novel Myxoma Virus C7-Like Host Range Factor That Enabled a Species Leap from Rabbits to Hares

**DOI:** 10.1128/mbio.03461-21

**Published:** 2022-03-30

**Authors:** Ana Águeda-Pinto, Simona Kraberger, Anne Everts, Ami Gutierrez-Jensen, Honor L. Glenn, Kevin P. Dalton, Ana Podadera, Francisco Parra, Monica Martinez-Haro, José Alberto Viñuelas, Arvind Varsani, Grant McFadden, Masmudur M. Rahman, Pedro J. Esteves

**Affiliations:** a CIBIO, Centro de Investigação em Biodiversidade e Recursos Genéticos, InBIO Laboratório Associado, Campus de Vairão, Universidade do Porto, Vairão, Portugal; b Departamento de Biologia, Faculdade de Ciências, Universidade do Porto, Porto, Portugal; c BIOPOLIS Program in Genomics, Biodiversity and Land Planning, CIBIO, Campus de Vairão, Vairão, Portugal; d Center for Immunotherapy, Vaccines and Virotherapy (CIVV), The Biodesign Institute, Arizona State University, Tempe, Arizona, USA; e The Biodesign Center for Fundamental and Applied Microbiomics, Center for Evolution and Medicine, School of Life Sciences, Arizona State University, Tempe, AZ , USA; f Instituto Universitario de Biotecnología de Asturias, Departamento de Bioquímica y Biología Molecular, Edificio Santiago Gascón, Universidad de Oviedo, Campus El Cristo, Oviedo, Spain; g Instituto Regional de Investigación y Desarrollo Agroalimentario y Forestal (IRIAF), CIAG del Chaparrillo, Ciudad Real, Spain; h Structural Biology Research Unit, Department of Integrative Biomedical Sciences, University of Cape Town, Observatory, Cape Town, South Africa; i CITS—Centro de Investigação em Tecnologias da Saúde, IPSN, CESPU, Gandra, Portugal; University of Utah; Duke University School of Medicine

**Keywords:** myxoma virus, leporids, species leap, host range, M159 protein, *Poxviridae*, C7 family

## Abstract

Myxoma virus (MYXV) is naturally found in rabbit *Sylvilagus* species and is known to cause lethal myxomatosis in European rabbits (Oryctolagus cuniculus). In 2019, an MYXV strain (MYXV strain Toledo [MYXV-Tol]) causing myxomatosis-like disease in Iberian hares (Lepus granatensis) was identified. MYXV-Tol acquired a recombinant region of ∼2.8 kb harboring several new genes, including a novel host range gene (*M159*) that we show to be an orthologous member of the vaccinia virus C7 host range family. Here, to test whether M159 alone has enabled MYXV to alter its host range to Iberian hares, several recombinant viruses were generated, including an MYXV-Tol ΔM159 (knockout) strain. While MYXV-Tol underwent fully productive infection in hare HN-R cells, neither the wild-type MYXV-Lau strain (lacking M159) nor vMyxTol-ΔM159 (deleted for M159) was able to infect and replicate, showing that the ability of MYXV-Tol to infect these cells and replicate depends on the presence of *M159*. Similar to other C7L family members, *M159* was shown to be expressed as an early/late gene but was translocated into the nucleus at later time points, indicating that further studies are needed to elucidate its role in the nucleus. Finally, in rabbit cells, the M159 protein did not contribute to increased replication but was able to upregulate the replication levels of MYXV in nonpermissive and semipermissive human cancer cells, suggesting that the M159-targeted pathway is conserved across mammalian species. Altogether, these observations demonstrate that the M159 protein plays a critical role in determining the host specificity of MYXV-Tol in hare and human cells by imparting new host range functions.

## INTRODUCTION

The ability of any virus to jump into a new host, successfully replicate, cause overt disease, and be transmitted to another individual of the new host species is governed by different molecular interactions between the host and the virus (1, 2). To begin addressing the fundamental questions mediating virus cross-species transmission, it is crucial to study the biological and mechanistic underpinnings driving such events using a well-established model system. The most extensively documented field model of pathogen evolution following a species jump is the introduction of the lethal myxoma virus (MYXV) into European rabbit (Oryctolagus cuniculus) populations in Australia and Europe as a biological control agent (3). The host range genes of MYXV evolved in the natural *Sylvilagus* hosts, yet when MYXV first encountered naive European rabbit populations, the virus immediately (i.e., with no required adaptation in the new host) caused an outbreak of a lethal disease called myxomatosis. In fact, when the highly virulent Standard Laboratory strain (SLS) was introduced in Australia and the South American strain of MYXV (Lausanne [Lau] strain) was introduced in France, the case fatality rates of infected rabbits were estimated to be ∼99% (4). In both cases, attenuated MYXV strains came to dominate field populations within a few years, allowing infected rabbits to survive longer and increasing the probability of transmission from skin lesions by arthropod vectors like mosquitoes (5, 6). The importance of MYXV and European rabbit interactions is very well known to the field of emerging infectious diseases since it provides an outstanding model to study dynamic host-pathogen interactions in the wild.

Recently, we characterized for the first time the full genome of recombinant MYXV variants (MYXV strain Toledo [MYXV-Tol], also previously described as ha-MYXV Tol08-18) capable of causing myxomatosis in a *Lepus* species (7–9). MYXV-Tol has high similarity to the MYXV-Lau strain previously released in Europe, with the exception of three disrupted genes (*M009L*, *M036L*, and *M152R*) plus a recombinantly derived cassette (recTol) of ∼2,800 bp from an unknown/unsampled poxvirus donor genome, which, based on the arrangement of the gene sequences, most closely resembles that from ungulate-associated poxviruses. The new recombinant cassette found in MYXV-Tol encodes a known poxviral structural protein (M157), a thymidine kinase (M158), a C7-like (C7L) host range protein (M159), and a poly(A) polymerase subunit (M160), which are most closely similar, but not identical, to the MYXV *M060R*, *M061R*, *M064R*, and *M065R* genes, respectively (7, 8). Analyses show that the newly discovered M159 protein encoded by MYXV-Tol has a high pairwise identity to the previously described MYXV host range proteins M064R and M062R that belong to the C7L family of host range factors (7, 9).

One of the most critical poxvirus host range factors belongs to the family of host-interactive viral proteins that share sequence similarity with the vaccinia virus (VACV) C7 protein, known to have major roles in pathogenicity (10). Sterile alpha motif domain-containing 9 (SAMD9), a large cytoplasmic protein with diverse functions, including antiviral, antineoplastic, and stress-responsive properties, is a key host target of many C7L viral proteins (10). In most mammalian poxviruses, at least one copy of a member of the C7L host range superfamily can be found encoded in the genome (11). Wild-type MYXV carries three tandem C7L genes that were probably derived from two distinct duplication events: *M062*, *M063*, and *M064* (10). Investigations on the roles of these host range genes have shown that only *M062* can be a substitute for VACV C7 in overcoming host range restriction by binding directly to SAMD9, while M063 facilitates only the latter interaction (12, 13). On the other hand, M064 does not exhibit any known host range properties but acts as a virulence factor that controls the kinetics of MYXV infection both *in vitro* and *in vivo* (14). The presence of this C7L protein in the recombinant region of MYXV-Tol prompted us to hypothesize that this newly acquired viral factor has enabled MYXV to alter its host range from rabbits to hares and caused the myxomatosis-like pathogenesis in hares.

Here, we report our study of the role of the M159-encoded protein as an MYXV-Tol virulence factor in cultured cells of hare, rabbit, and human origins by examining the phenotypes of different recombinant MYXVs *in vitro*, including a targeted MYXV-Tol M159 knockout (vMyxTol-ΔM159) and an MYXV-Lau strain expressing the novel M159 protein (vMyxLau-V5M159) alone. Moreover, a V5-tagged M159 protein was constructed to characterize the expression patterns and localization of this viral host range protein in cultured cells. The data show that the M159 protein is required for productive MYXV replication in hare HN-R cells and improves the replication of MYXV in nonpermissive or semipermissive human cancer cells, thus providing a platform for a better understanding of poxvirus host range and cross-species transmission.

## RESULTS AND DISCUSSION

**FIG 1:**
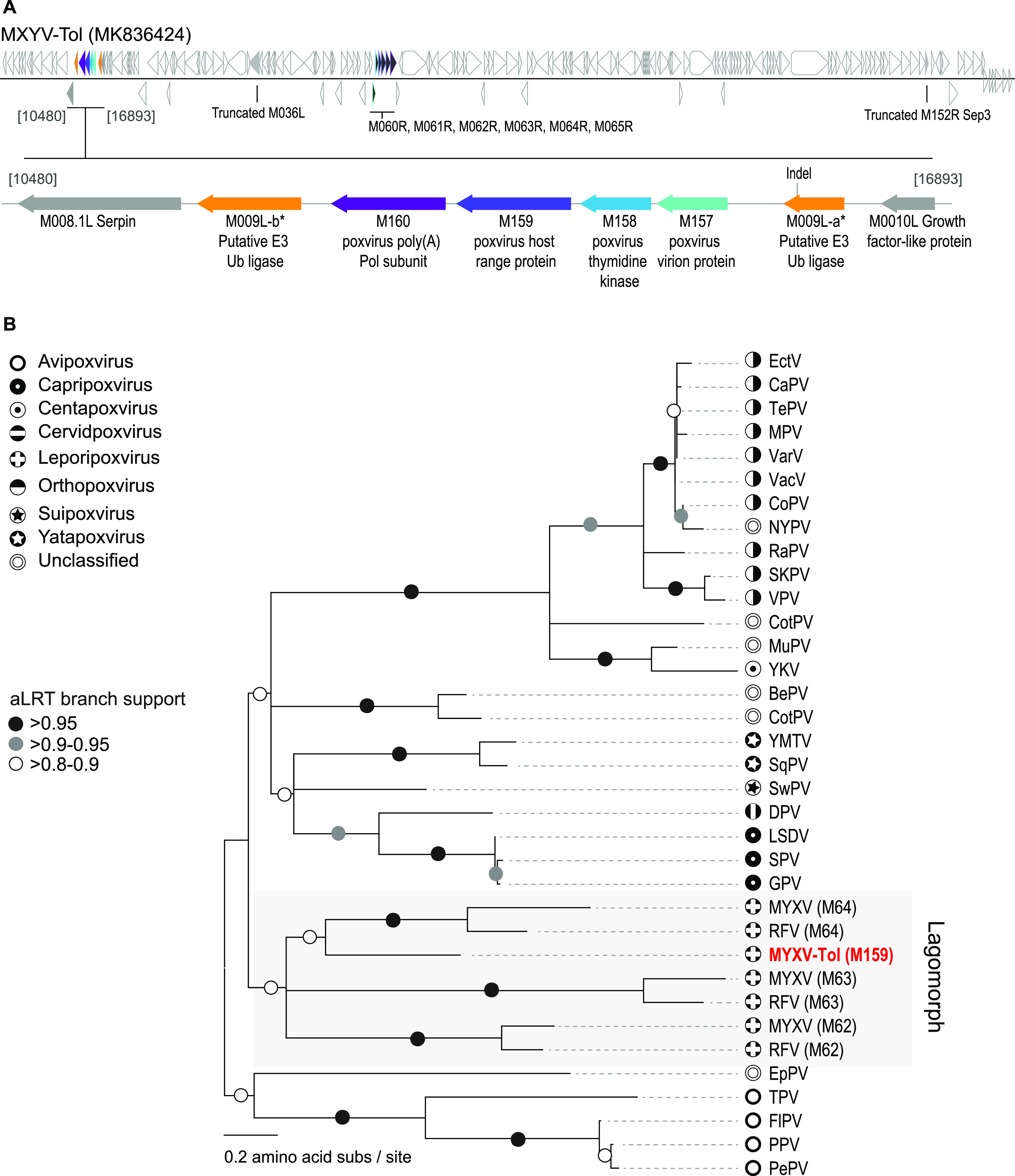
The newly discovered M159 protein from MYXV-Tol belongs to the C7 superfamily of host range proteins. (A) Representation of the genome organization of MYXV-Tol (GenBank accession no. MK836424). Orange shows the disrupted *M009L* gene. Blue and purple ORFs show the new gene “cassette” identified and the location of the *M157*, *M158*, *M159*, and *M160* genes in MYXV-Tol. Ub, ubiquitin; Pol, polymerase. (B) Phylogenetic analysis of C7-like members from 23 different poxviruses. EctV, Ectromelia virus; CaPV, Capripox virus; TePV, Taterapox virus; MPV, Monkeypox virus; VarV, Variola virus; VacV, Vaccinia virus; CoPV, Cowpox virus; NYPV, NY_014 poxvirus; RaPV, Raccoonpox virus; SKPV, Skunkpox virus; VPV, Volepox virus; CotPV, Cotia virus; MuPV, Murmansk poxvirus; YKV, Yokapox virus; BePV, BeAn 58058 virus; YMTV, Yaba monkey tumor virus; SqPV, Squirrelpox virus; SwPV, Swinepox virus; DPV, Deerpox virus; LSDV, Lumpy skin disease virus; SPV, Sheeppox virus; GPV, Goatpox virus; EpPV, Eptesipox virus; TPV, Turkeypox virus; FIPV, Flamingopox virus; PPV, Pigeonpox virus; PePV, Penguinpox virus.

Since the autumn of 2018, hundreds of Iberian hares from the Iberian Peninsula, Europe, have died as a result of a viral infection with a newly discovered MYXV strain, named MYXV-Tol here (7, 15). This MYXV strain has a high nucleotide identity to previously described strains such as MYXV-Lau, with the exception of a unique “cassette” of genes. Interestingly, some studies highlight that Iberian hares have been in contact with MYXV (or an antigenically similar virus) since at least the 1990s (16) without an outbreak occurring, suggesting that the recent acquisition of this unique cassette of genes (recombination event between MYXV and an unknown poxvirus) allowed MYXV to cross the species barrier, causing disease in these animals. The predicted functions of the four newly acquired viral proteins [virion structural protein, thymidine kinase, C7L host range member, and poly(A) polymerase subunit] found in the recombinant region are in accordance with the gene order arrangement found in the ∼57,500-bp region of other poxviruses (7, 9). However, given the fact that this cassette is apparently inverted in MYXV-Tol and that the predicted encoded proteins show relatively low similarity to the existing gene members in MYXV-Lau, we have assigned new viral open reading frame (ORF) numbers, which we refer to as M157 for the virion structural protein, M158 for the thymidine kinase, M159 for the C7L host range factor, and M160 for the poly(A) polymerase subunit (Fig. 1A).

### M159 is a new member of the C7L family of poxviral host range genes.

VACV C7 family proteins are present in almost all poxviruses that infect mammalian species, including MYXV, Yaba-like diseases virus (YLDV), swinepox virus (SWPV), and sheeppox virus (SPPV) (10, 11, 17). As shown in Fig. 1B, the new MYXV-Tol *M159* gene found in the recombinantly derived region encodes a predicted member of the C7L family of host range factors. Previous reports show that the recombinant cassette that was inserted within the *M009L* gene of MYXV-Tol originated from a still-unknown donor poxvirus that shares an ancestral lineage with capripoxviruses, cervidpoxviruses, suipoxviruses, and yatapoxviruses. Yet it is interesting to observe that the newly identified M159 protein has a higher pairwise identity to the C7L family of host range proteins that are found in leporipoxviruses. In fact, and as shown in Fig. 1B, the M159 protein is more closely related to the M064 proteins from MYXV and rabbit fibroma virus (RFV), sharing 41.8% and 39.9% pairwise amino acid identities, respectively. M159 also shows a high degree of similarity to M063 and M062 from MYXV and RFV, with pairwise identities that range from 25.3 to 44.3% (Fig. 1B).

**FIG 2:**
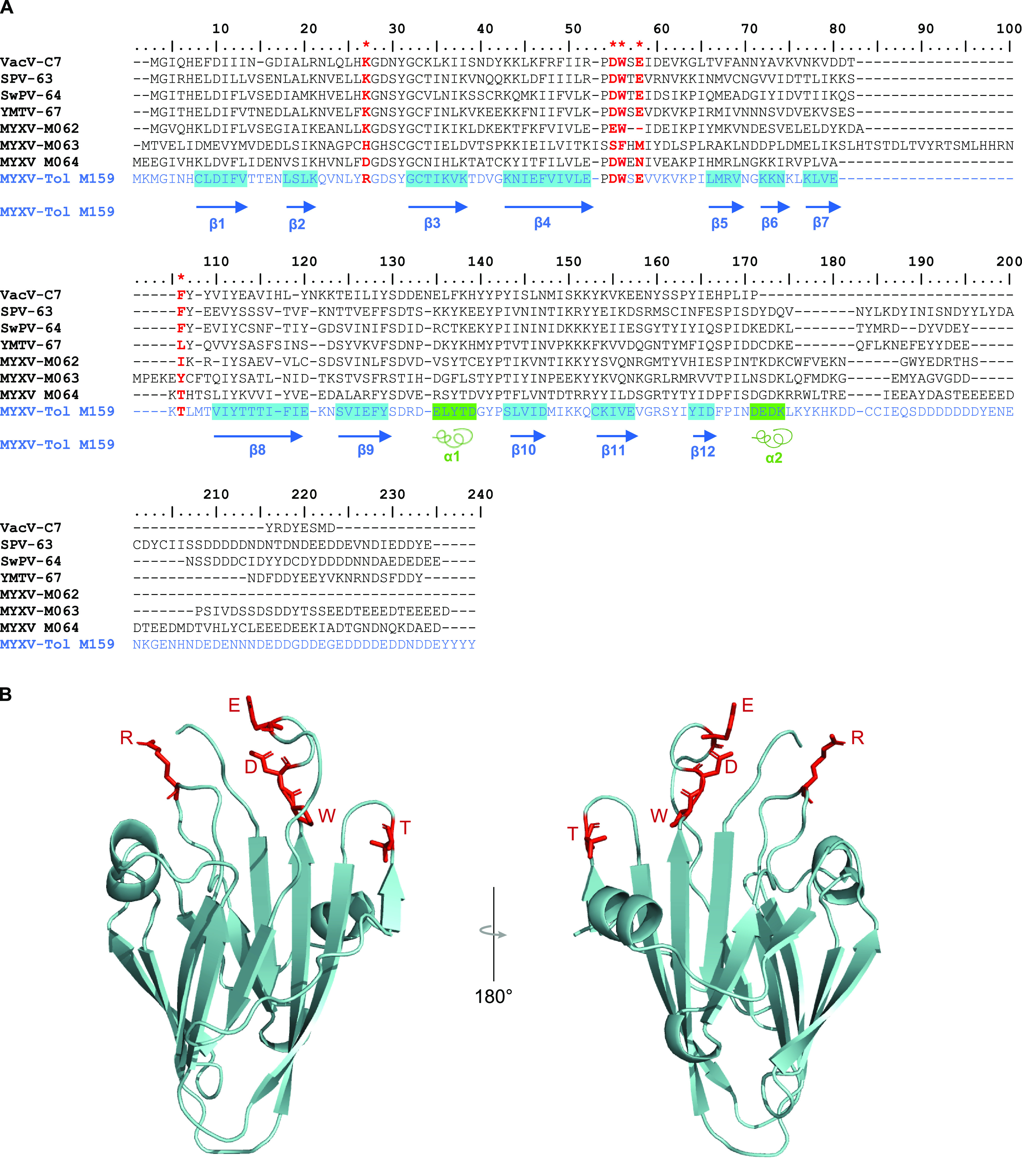
M159 adopts the conserved structure of the C7 protein family. (A) Amino acid sequence comparison of different members of the C7-like superfamily of host range genes, including VACV C7; SPPV-63; SWPV-64; YLDV-67; MYXV M062, M063, and M064; and MYXV-Tol M159. The 12-stranded antiparallel β-sandwich (blue boxes) and the two short α-helices (green boxes) from MYXV-Tol M159 are highlighted. The five VACV C7 conserved residues (K24, D51, W52, E54, and F79) critical for binding to SAMD9 are indicated by red asterisks. (B) Homology-modeled structure of the M159 protein. The 12-stranded antiparallel β-sandwich and two α-helices are shown in light green. The five VACV C7 conserved residues (K24, D51, W52, E54, and F79) are shown in red.

Despite the fact that C7L family members frequently have low sequence identity to each other, their tertiary structures tend to be relatively conserved (18). This family of proteins has adopted a compact β-sandwich fold with approximate dimensions of 45 by 35 by 30 Å that consists mainly of two curved layers, each comprising a six-stranded antiparallel β-sheet (18). Therefore, we carried out homology modeling of the newly identified M159 structure using MYXV M064 and VACV C7 as the templates (Protein Data Bank accession no. 5CZ3B and 5CYWB, respectively). The M159 protein homology model shows the conserved structure of the C7L protein family with a modeled 12-stranded antiparallel β-sandwich wrapped in two short α-helices (Fig. 2B). On one side, the M159 protein has a layer of strands β1, β2, β4, β7, β8, and β12 and a second layer of strands β3, β5, β6, β9, β10, and β11. Moreover, the M159 model also includes two α-helices (α1 and α2), with helix α1 linking strands β9 and β10 and helix α2 being located at the C terminus (Fig. 2B). We predict that, like other C7L members, M159 functions via host-derived binding partners, but the sequence divergence within the C7L family is sufficiently large that it is difficult to model how different the putative host target(s) of M159 might be, in terms of its closest known poxvirus gene relatives.

Previous reports suggest that the functional core for the C7L family is the conserved β-sandwich (10). Structure-guided mutagenesis studies on VACV C7 identified residues K24, D51, W52, E54, and F79 as being critical for binding to SAMD9 and, therefore, viral replication (18). These residues are located in three loops clustered on one edge of the β-sandwich and are characterized by functionally important negatively charged, positively charged, and hydrophobic residues, forming a unique “three-fingered molecular claw.” Interestingly, this claw is not conserved in the MYXV M063 and M064 proteins, two C7L family members that do not antagonize SAMD9 (18). Analysis of the five conserved C7L residues at the corresponding positions of the M159 protein show that only D51, W52, and E54 are conserved (Fig. 2A). Interestingly, in the corresponding position of K24, the M159 protein has an arginine (R) that, like lysine (K), is positively charged. However, in the corresponding position of F79, the M159 protein has a threonine (T), which, contrarily to phenylalanine (F), is not a hydrophobic residue (Fig. 2A). The replacement of a hydrophobic residue for a polar residue (T) in the three-fingered molecular claw responsible for binding to SAMD9 might suggest a lower affinity for SAMD9 or a different conformation of this claw in the M159 protein and, therefore, a different binding partner or function compared to the ones observed for VACV C7 and MYXV M062.

### M159 is essential for the replication of MYXV-Tol in cultured hare cells.

Regarding the expansion of the host range, it is likely that the acquisition of new genes involved in immunomodulation or host range functions in specific cell types would have a preponderant role in this apparent species leaping of MYXV-Tol. Moreover, given that the *M159* gene was acquired from a currently unknown “donor” poxvirus with a different repertoire of antiviral response pathways, we speculated that this new host range protein is capable of differentially modulating the antiviral response of hare versus rabbit cells.

To elucidate the role of the *M159* gene and/or gene product during MYXV infection, we constructed a knockout virus from the wild-type MYXV-Tol backbone in which only the *M159* gene was disrupted by a gene cassette encoding reporter green fluorescent protein (GFP) and tandem dimer tomato fluorescent protein (TdT) under the control of an early-late promoter (synE/L) and a late p11 promoter, respectively (vMyxTol-Δ159). To understand the importance of the *M159* gene alone, a V5-tagged *M159* gene (under the control of its native promoter) was inserted between the *M135L* and *M136L* gene loci of the MYXV-Lau backbone along with the GFP gene under the control of a synE/L promoter (vMyxLau-V5M159). In addition, to understand the importance of the remaining genes of the recTol region relative to the *M159* gene alone, we constructed two recombinant viruses where the entire 2.7-kb cassette was inserted into two different regions of MYXV-Lau, one between the *M135L* and *M136L* gene loci (vMyxLau-recTol) and another one within the *M009L* gene (Δ*M009L*), replicating the position of the recTol region in wild-type MYXV-Tol (vMyxLau-ΔM009L+recTol). All these viruses were studied and compared to wild-type MYXV-Lau and MYXV-Tol, both expressing a GFP and TdT gene cassette under the control of a synE/L promoter and a p11 late promoter, respectively (vMyxLau and vMyxTol). This reporter gene cassette was inserted between the conserved intergenic regions of the *M135* and *M136* genes. Table 1 illustrates the characteristics of all recombinant viruses used in this study.

**TABLE 1:** Summary of the recombinant viruses used in this study^*a*^

Recombinant virus	Backbone	Insertion(s)	Disrupted gene	Virus abbreviation
vMyxTol-TdT-GFP	MYXV-Tol	TdT, GFP		vMyxTol
vMyxTol-ΔrecTol-GFP	MYXV-Tol	GFP	ΔrecTol region	vMyxTol-ΔrecTol
vMyxTol-ΔM159-TdT-GFP	MYXV-Tol	TdT, GFP	ΔM159	vMyxTol-ΔM159
vMyxTol-V5tagM159-GFP	MYXV-Tol	V5-tagged M159, GFP		vMyxTol-V5M159
vMyxLau-TdT-GFP	MYXV-Lau	TdT, GFP		vMyxLau
vMyxLau-ΔM009-TdT-recTol-GFP	MYXV-Lau	TdT, GFP, recTol region	ΔM009	vMyxLau-ΔM009+recTol
vMyxLau-recTol-TdT-GFP	MYXV-Lau	TdT, GFP, recTol region		vMyxLau-recTol
vMyxLau-V5tagM159-GFP	MYXV-Lau	V5-tagged M159, GFP		vMyxLau-V5M159

a TdT, late p11 promoter; GFP, early-late promoter.

**FIG 3:**
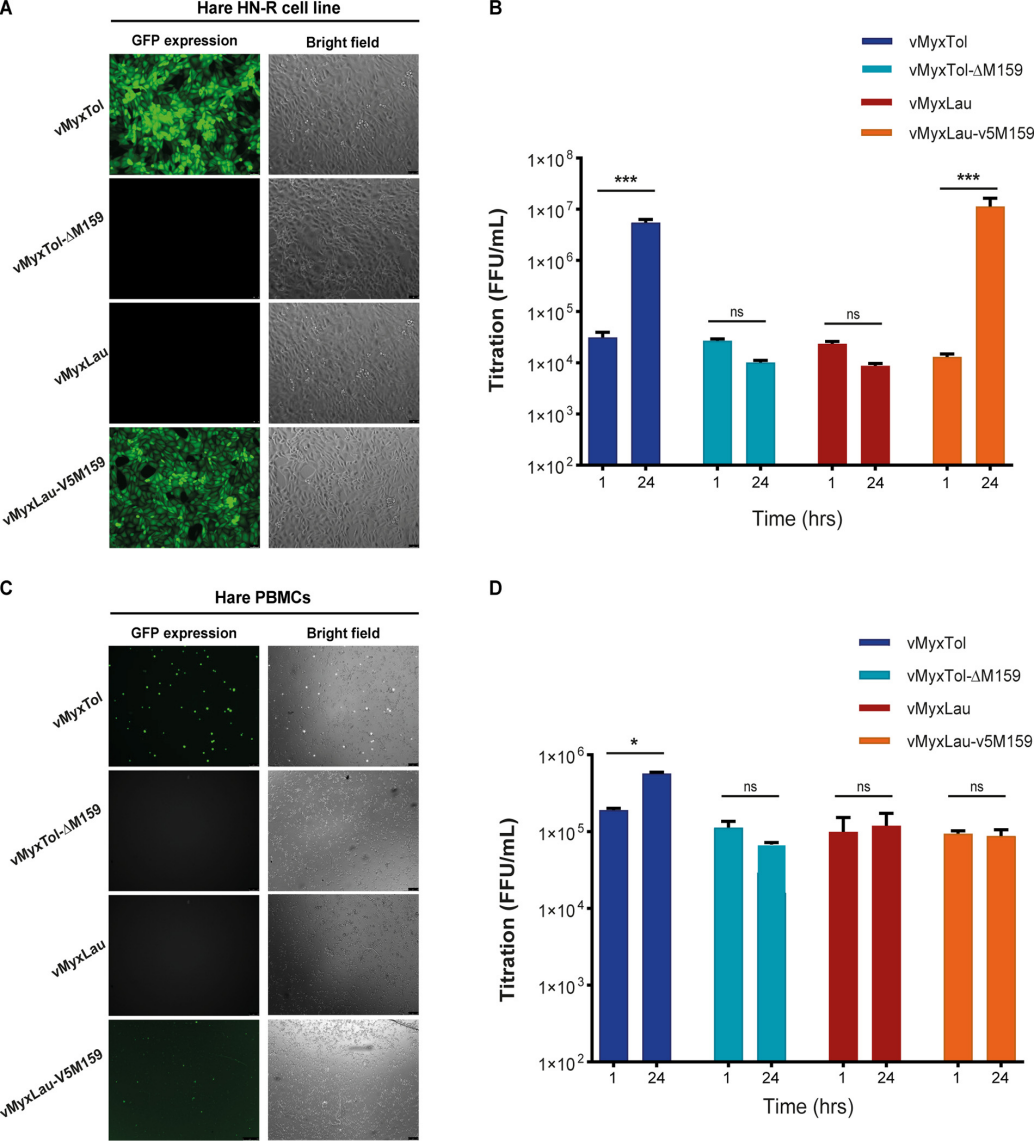
M159 is required for MYXV-Tol replication in hare PBMCs and the hare HN-R cell line. (A) Fluorescence microscopy images of infected hare HN-R cells. The cells were infected with vMyxTol, vMyxTol-Δ159, vMyxLau, and vMyxLau-V5M159 at an MOI of 1, and the images were taken using an inverted fluorescence microscope at a ×5 magnification at 24 hpi. (B) Proliferation of different recombinant MYXVs in the hare HN-R cell line. The cells were infected with the indicated viruses at an MOI of 1 for 1 h and 24 h. The virus titers were investigated following serial dilutions onto rabbit RK13 cells. The results are expressed as means ± standard errors (SE) from three independent experiments. *, *P* value of <0.05. (C) Fluorescence microscopy images of infected hare PBMCs. The cells were infected with vMyxTol, vMyxTol-Δ159, vMyxLau, and vMyxLau-V5M159 at an MOI of 10, and the images were taken using an inverted fluorescence microscope at a ×5 magnification at 24 hpi. (D) Proliferation of different recombinant MYXVs in hare PBMCs. The cells were infected with the indicated viruses at an MOI of 10 for 1 h and 24 h. The virus titers were investigated following serial dilutions onto rabbit RK13 cells. Significant differences for experiments were analyzed using two-way analysis of variance (ANOVA) followed by Dunnett’s test using Prism 6 (GraphPad Software, Inc.), and the results are expressed as means ± SE from three independent experiments. ns, nonsignificant; *, *P* < 0.05; ***, *P* < 0.001 (compared to 1 hpi).

When MYXV recombinant viruses were tested in two different hare cell types, the hare HN-R cell line and primary hare peripheral blood mononuclear cells (PBMCs), these test cells engaged similarly in supporting dramatically different levels of viral infection that reflect the essential nature of the M159 gene. As expected, MYXV-Tol (vMyxTol) was able to infect both cell types (Fig. 3A and C), whereas infection was blocked in wild-type MYXV-Lau (vMyxLau) (Fig. 3A and C). Remarkably, knockout of the M159 gene alone in the MYXV-Tol backbone (vMyxTol-Δ159) resulted in an abortive replication cycle in the hare HN-R cell line and hare PBMCs, as there was no increase in the vMyxTol-Δ159 titer after 24 h postinfection (hpi) (Fig. 3B and D). Since both hare cell types reached higher progeny virus titers after 24 hpi with vMyxTol, and disruption of M159 in the MYXV-Tol backbone resulted in abortive replication, this highly suggests that the M159 gene is critical for MYXV-Tol infection. Interestingly, while the insertion of the *M159* gene in the MYXV-Lau backbone (vMyxLau-V5M159) enabled infection in hare HN-R cells, as vMyxLau-V5M159 showed efficient virus infection and an increase in the virus titer after 24 hpi (Fig. 3A and B), infection of hare PBMCs with vMyxLau-V5M159 did not lead to an increase in the virus titer after 24 hpi (Fig. 3D). In fact, infection of hare PBMCs by vMyxLau-V5M159 led to early viral gene expression (GFP expression in Fig. 3C), but late viral expression was absent (not shown), suggesting that in hare PBMCs, M159 alone is not enough to rescue vMyxLau and that primary PBMCs might have activated innate antiviral pathways that block the replication of vMyxLau-V5M159. Interestingly, in the hare HN-R cell line, the three viruses that have MYXV-Lau as the backbone (vMyxLau-V5M159, vMyxLau-recTol, and vMyxLau-ΔM009+recTol) exhibited similar replication and progeny virus yields (see Fig. S1 in the supplemental material), suggesting that the disruption of the *M009L* gene with the recTol region does not alter MYXV replication and that the position of the recTol cassette does not affect MYXV infection of hare HN-R cells.

10.1128/mbio.03461-21.1 FIG S1 M159 is required for MYXV replication in hare HN-R cells. (A) Single-step growth curve of MYXV infection in hare HN-R cells. The cells were infected with vMyxTol, vMyxTol-Δ159, vMyxLau, vMyxLau-V5M159, vMyxLau-ΔM009+recTol, and vMyxLau-recTol at an MOI of 1 and then collected at 0, 8, 24, 48, and 72 hpi. The virus titers are representative of results from three independent experiments and were determined in triplicate following serial dilution onto rabbit RK13 cells. Differences at a *P* value of <0.05 were considered significant. (B) Fluorescence microscopy images of infected HN-R cells. The cells were infected with vMyxTol, vMyxTol-Δ159, vMyxLau, vMyxLau-V5M159, vMyxLau-ΔM009+recTol, and vMyxLau-recTol at an MOI of 1, and the images were taken using an inverted fluorescence microscope using a lens with a ×5 magnification at 24 hpi. Download FIG S1, PDF file, 2.0 MB. Copyright © 2022 Águeda-Pinto et al. 2022 Águeda-Pinto et al. https://creativecommons.org/licenses/by/4.0/ This content is distributed under the terms of the Creative Commons Attribution 4.0 International license.

### M159 is essential for viral late protein synthesis in hare cells.

**FIG 4:**
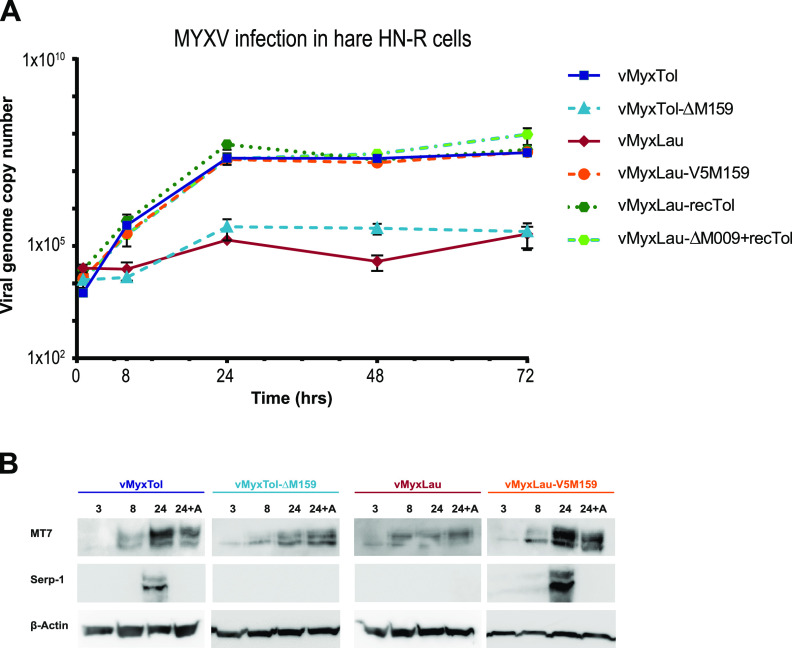
M159 is essential for MYXV transition into late expression in hare HN-R cells. (A) Infection with vMyxLau and M159 knockout virus (vMyxTol-Δ159) in hare HN-R cells. HN-R cells were infected with vMyxTol, vMyxTol-Δ159, vMyxLau, vMyxLau-V5M159, vMyxLau-ΔM009+recTol, and vMyxLau-recTol at an MOI of 1. At the indicated time points (0, 8, 24, and 48 hpi), cell lysates were harvested for DNA extraction, followed by qPCR targeting the MYXV M053 gene. Differences among means from three independent experiments were investigated using two-way ANOVA followed by Dunnett’s test using Prism 6 (GraphPad Software, Inc.). Differences at a *P* value of <0.05 were considered significant. (B) Western blot analyses of early and late viral gene expression after infection with vMyxTol, vMyxTol-Δ159, vMyxLau, and vMyxLau-V5M159. HN-R cells were pretreated for 1 h with AraC (40 μg/mL) (A- AraC treatment) or not pretreated, followed by infection at an MOI of 5. At the indicated time points (3, 8, and 24 hpi), cell lysates were harvested for Western blotting. Early/late gene expression (M-T7) and late gene expression (Serp-1) were compared between the viruses, with actin used as a control. The molecular sizes are 35 kDa for M-T7, 55 kDa for Serp-1, and 42 kDa for actin. The results shown are representative of data from two independent experiments.

The microscopy analyses of infected cultures show that vMyxLau or vMyxTol-ΔM159 induced only a barely detectable level of GFP expression and no TdT expression in hare HN-R cells (Fig. S1). In contrast, vMyxTol, vMyxLau-V5M159, vMyxLau-recTol, and vMyxLau-ΔM009+recTol productively infected HN-R hare cells after 24 hpi, as can be seen by the robust expression of the late reporter protein TdT (Fig. 3A and Fig. S1). We further confirmed the importance of the M159 protein for MYXV replication in the hare HN-R cell line by monitoring the viral genomic copy numbers after infection at different time points (Fig. 4A). The quantitative PCR (qPCR) results show that both vMyxLau and vMyxTol-ΔM159 genomic DNAs are detectable in hare cells at 8 hpi; however, at later time points, viral genome amplification was significantly reduced compared to that of vMyxTol. Again, the presence of the complete recTol region and/or the *M159* gene locus in the MYXV-Lau backbone shows the same trend for viral replication as that for vMyxTol (Fig. 4A).

In contrast to many other DNA viruses, poxvirus DNA replication takes place in cytoplasmic viral factories (19). Upon infection, poxviruses exhibit tight regulation of a gene expression cascade that results from the genes being regulated by promoters that are transcribed at early, intermediate, and/or late times of infection (19, 20). In infection by VACV, following virion binding to the cell surface and internalization, there is a release of the virion core into the cytoplasm, followed by the transcription of early viral mRNAs that typically encode products required for immune evasion, core uncoating, the release of genomic DNA, and the initiation of DNA synthesis (21). The beginning of viral DNA replication coincides with a switch to the transcription of intermediate and then late mRNAs whose products are required for virion assembly (19, 20). Finally, viral genome replication and the assembly of progeny virus particles occur in cytoplasmic viral factories (22, 23). In this study, we have shown that a block in wild-type MYXV-Lau replication exists in hare HN-R cells when the M159 protein is not present (i.e., cells infected with vMyxTol-ΔM159 and vMyxLau) (Fig. 4A). To evaluate when this block occurs in hare HN-R cells, the expression of known early and late MYXV proteins was tested by Western blotting (24, 25). We infected hare HN-R cells with vMyxTol, vMyxTol-ΔM159, vMyxLau, and vMyxLau-V5M159 at a high multiplicity of infection (MOI) (MOI = 5) and left the cells untreated or added cytosine arabinoside (AraC) (40 μg/mL), an inhibitor of poxviral DNA replication and late protein synthesis, for 1 h following virus adsorption. Infected cell extracts were then immunoblotted using anti-M-T7, a strongly expressed MYXV early gene product, and anti-Serp-1, an MYXV late gene product (24, 25). The early M-T7 protein was detectable by as early as 3 hpi and at 24 hpi regardless of the virus used, in the presence or absence of AraC (Fig. 4B). Late Serp-1 was detectable at 24 hpi in hare HN-R cells infected by vMyxTol and vMyxLau-V5M159, except when DNA synthesis was blocked by the addition of AraC. On the other hand, Serp-1 was not detectable at any time point evaluated for vMyxTol-ΔM159 or vMyxLau-infected hare cells (Fig. 4B). These results reinforce the above-described demonstration that infection by vMyxTol-Δ159 and vMyxLau undergoes an early block in viral replication, with M159 playing a critical role in the transition to late stages of infection and replication in hare HN-R cells. Moreover, while there was evidence for the existence of viral factories in hare HN-R cells infected by vMyxTol and vMyxLau-V5M159 (arrows in Fig. S2), the same was not observed after infection by vMyxTol-Δ159 or vMyxLau (Fig. S2). Overall, while early gene expression remained unaltered in cell infections by all recombinant MYXVs tested, the absence of M159 in MYXV prevented viral factory formation in hare HN-R cells and thus culminated in defective late protein production and viral DNA replication. Altogether, these results confirm that the *M159* gene product is necessary and sufficient for the successful replication of MYXV in hare HN-R cells.

10.1128/mbio.03461-21.2 FIG S2 Viral factories in hare HN-R cells infected with vMyxTol or vMyxLau-V5M159. HN-R cells grown on glass coverslips were infected with vMyxTol, vMyxTol-ΔM159, vMyxLau, and vMyxLau-V5M159 at an MOI of 5. After 24 hpi, cells were fixed, permeabilized, stained, and imaged by confocal microscopy. DNA in the nucleus and viral factory was labeled with DAPI (blue). Infection was tracked by GFP expression (green). White arrows indicate the presence of viral factories. Download FIG S2, PDF file, 2.0 MB. Copyright © 2022 Águeda-Pinto et al. 2022 Águeda-Pinto et al. https://creativecommons.org/licenses/by/4.0/ This content is distributed under the terms of the Creative Commons Attribution 4.0 International license.

### M159 is expressed as an early/late product that is translocated into the nucleus at late time points in virus-infected hare cells.

Previous studies indicate that the other MYXV C7L members are expressed as early/late genes (12–14). Given the higher similarity of *M159* to MYXV C7L genes (Fig. 1B), we tested whether M159 is also expressed as an early/late product. For this, a recombinant MYXV-Tol strain was constructed bearing V5-tagged M159 (vMyxTol-V5M159) inserted between the *M135L* and *M136L* gene loci and expressed GFP under the control of a poxvirus early/late promoter (Table 1). Given that vMyxTol-V5M159 has two *M159* gene copies, virus replication was tested in the hare HN-R cell line and compared with that of wild-type MYXV-Tol (vMyxTol). Both viruses show similar transmission kinetics and progeny virus titers after 96 hpi in HN-R cells (Fig. S3). A time course study of M159 protein synthesis was conducted by infecting HN-R cells at an MOI of 5 in the presence or absence of AraC. Similar studies were also conducted with vMyxLau-V5M159 described above (Fig. S4).

10.1128/mbio.03461-21.3 FIG S3 Additional copies of the *M159* gene in vMyxTol do not alter MYXV infection in hare HN-R cells. The cells were infected with vMyxTol and vMyxTol-V5M159 at an MOI of 3 and then collected at 0, 6, 12, 24, 48, 72, and 96 hpi. The virus titers are representative of results from three independent experiments and were determined in triplicate following serial dilution onto RK13 cells. Significant differences among means were determined using two-way ANOVA followed by Dunnett’s test using Prism 6 (GraphPad Software, Inc.). Differences at a *P* value of <0.05 were considered significant. Download FIG S3, PDF file, 0.08 MB. Copyright © 2022 Águeda-Pinto et al. 2022 Águeda-Pinto et al. https://creativecommons.org/licenses/by/4.0/ This content is distributed under the terms of the Creative Commons Attribution 4.0 International license.

10.1128/mbio.03461-21.4 FIG S4 M159 in the MYXV-Lau background is expressed late and accumulates throughout the course of infection. HN-R cells were pretreated or not for 1 h with AraC, followed by infection with vMyxLau-V5M159 at an MOI of 5 in the presence or absence of AraC. At the indicated time points (1, 3, 8, 24, and 48 hpi), cell lysates were harvested for Western blotting. V5-tagged M159 was detected by probing with the anti-V5 antibody (top row), and actin was used as an internal control (bottom row). The molecular mass of actin is 42 kDa, and the expected molecular mass for the V5-tagged product is ∼25 kDa. Download FIG S4, PDF file, 0.5 MB. Copyright © 2022 Águeda-Pinto et al. 2022 Águeda-Pinto et al. https://creativecommons.org/licenses/by/4.0/ This content is distributed under the terms of the Creative Commons Attribution 4.0 International license.

**FIG 5:**
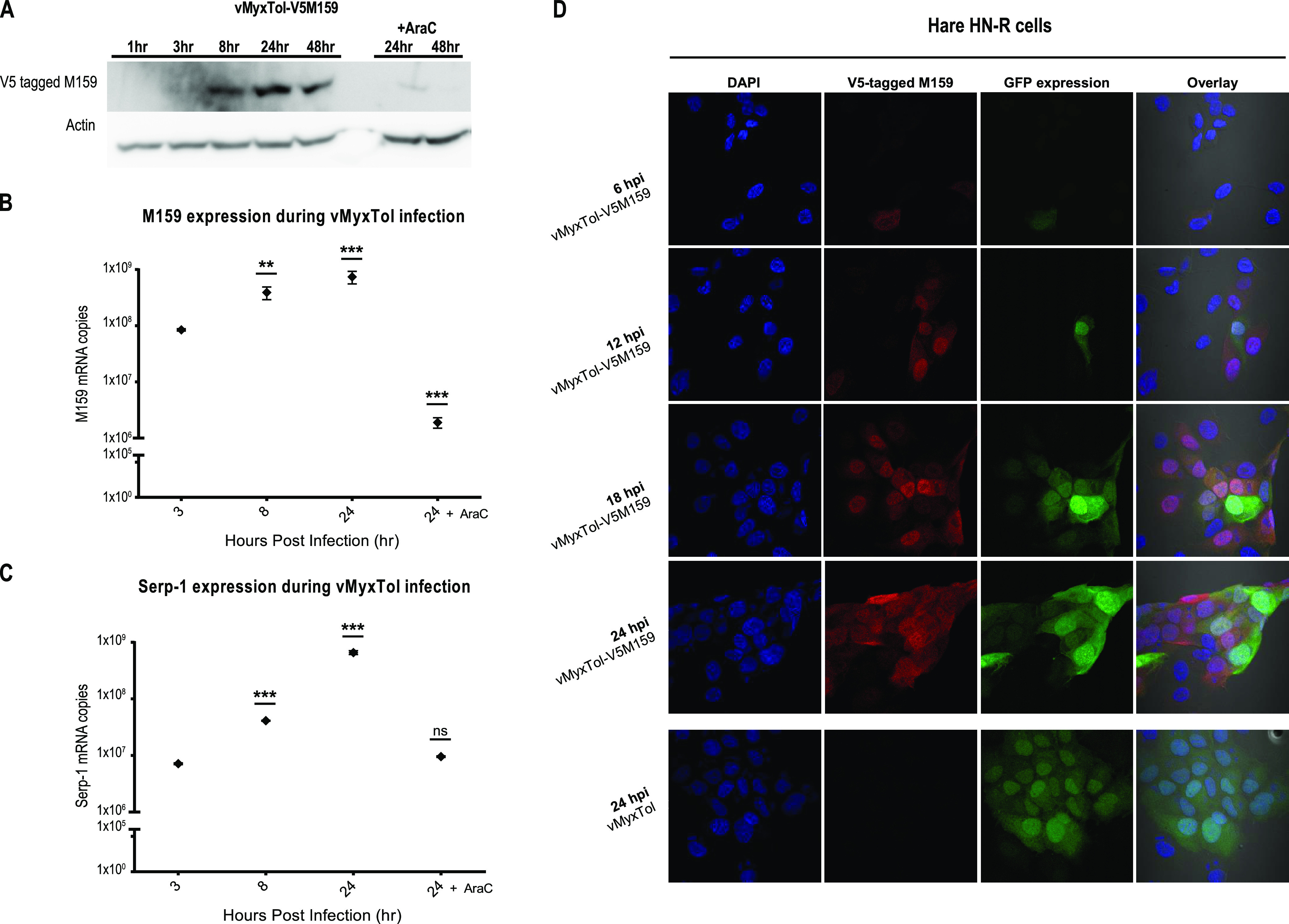
M159 is expressed as a late gene and moves into the nucleus at later time points of infection. (A) Western blot analysis of M159 expression during viral infection. HN-R cells were pretreated or not with AraC (40 μg/mL), followed by infection with vMyxTol-V5M159 at an MOI of 5. At the indicated time points (1, 3, 8, 24, and 48 hpi), cell lysates were harvested for Western blotting. V5-tagged M159 was detected with the anti-V5 antibody. The molecular mass of actin is 42 kDa, and the expected size for the V5-tagged product is ∼25 kDa. Three independent experiments were performed to confirm the obtained results. (B and C) RT-qPCR was used to investigate the expression of two MYXV genes, *M159* (B) and *Serp-1* (C). The comparative threshold cycle (*C_T_*) method was used to calculate and compare the relative levels of RNA. A control of HN-R cells pretreated with AraC for 1 h before infection and collected at 24 hpi was also used under the same conditions. Significant differences for the experiments in panels B and C were analyzed using two-way ANOVA followed by Dunnett’s test using Prism 6 (GraphPad Software, Inc.), and the results are expressed as means ± SE from three independent experiments. ns, nonsignificant; **, *P* < 0.01; ***, *P* < 0.001 (compared to 3 hpi). (D) M159 localizes to the nuclear and cytoplasmic compartments. HN-R cells grown on glass coverslips were infected with vMyxTol-V5M159 and vMyxTol (negative control) at an MOI of 1. After 6, 12, 18, and 24 hpi, cells were fixed, permeabilized, stained, and observed using confocal microscopy. DNA in the nucleus and viral factories were labeled with DAPI (blue). Infection was tracked by GFP expression (green) and V5-tagged M159 (red) using immunolabeling.

Contrary to what is observed for the three MYXV C7L members (12–14), the M159 protein was detected at high concentrations only at 8 hpi (Fig. 5A), and its levels were maintained until 48 hpi (Fig. 5A). However, following treatment with AraC, M159 protein levels were drastically reduced, with only a small amount of M159 being detected at 24 hpi (Fig. 5A). Given that AraC acts to block DNA synthesis, these results suggest that the *M159* gene has an early/late promoter but with the bulk of M159 being expressed as a late gene. To confirm these results, *M159* gene expression was analyzed by qPCR using primers and a probe that specifically target the mRNA product of this gene. Despite M159 protein levels starting to be easily detectable only at 8 hpi (Fig. 5A), *M159* mRNA can be detected at 3 hpi and increases over time, with maximum expression levels at 24 hpi (Fig. 5B). This difference between the protein and mRNA levels of *M159* could be due to the lower sensitivity of protein detection by Western blotting or the coincident early transcription of the M159 gene from an upstream promoter in the vMyxTol genome. The expression levels of *M159* and Serp-1 mRNAs were noticeably lower following treatment with AraC (Fig. 5B and C), suggesting once more that *M159* is expressed as an early/late gene, which would be consistent with its function in relieving an early block of viral replication.

We next examined the localization of M159 protein in virus-infected hare HN-R cells using confocal microscopy (Fig. 5D). When hare HN-R cells were infected with vMyxTol-V5M159, the expression of the V5-tagged M159 protein was detected after 6 hpi (Fig. 5D, row 1). At later time points (12, 18, and 24 hpi), M159 protein started to be detected in both the cytoplasmic and nuclear compartments of hare HN-R cells (Fig. 5D, rows 2 to 4). Interestingly, throughout the time course of the experiment, no V5-tagged M159 was detected in the viral factories. C7L family protein localization is significantly dependent upon interactions with host binding partners. The VACV C7 protein and some of its related host range proteins use their three-fingered molecular claw to bind and counteract cellular SAMD9. This cytoplasmic host cell protein is known to act as a major restriction factor in poxvirus infection, triggering an early block in infection, and is involved in the inhibition of viral mRNA translation (18, 26). The same has been shown for the MYXV M062 protein, which also binds and counteracts the action of SAMD9 (12). The data in this work indicate that like other C7L members, the M159 protein is also located in the cytoplasmic compartment during infection, suggesting a possible interaction with SAMD9. However, given the presence of M159 in the nucleus at later time points, it is possible that this protein might also have a specific function in this compartment. Nevertheless, the ability of M159 to bind to SAMD9 remains to be tested.

Over the past years, proteomic analyses, such as coimmunoprecipitation (co-IP) and mass spectrometry, have been foundational in identifying host factors that interact with C7L family members. However, as some interactions can be transient or cellular localization dependent, co-IP techniques may not always be ideal for interaction analyses. In this study, co-IP techniques that successfully identified the host binding partners of different MYXV proteins like M029 and M062 were used to try to identify V5-tagged M159 binding partners (12, 27). However, when using these techniques with vMyxTol-v5M159 and vMyxLau-v5M159, we were unable to identify a long-lived direct binding partner(s) for the M159 protein in hare HN-R cells. So far, the only known mechanism for the C7-like genes to confer a host range effect is through the inhibition of host SAMD9 (12, 13). However, there is no available *SAMD9* or *SAMD9L* sequence for hare species (*Lepus* sp.) in online databases, and our efforts to clone the hare SAMD9 sequence based on other mammalian SAMD9 sequences failed. In future studies, it will be important to either rule in or rule out hare SAMD9 and SAMD9L proteins and also continue to study transitory protein-protein interactions to elucidate the mechanism of M159 action involved in MYXV-Tol host range in hares.

### M159 is not essential for MYXV infection and replication in rabbit cells.

Recently, MYXV-Tol was also reported to be capable of causing myxomatosis in European rabbits (15). However, these reported cases of pathogenesis were less frequent in rabbits, suggesting a different susceptibility to MYXV-Tol among the lagomorphs. To compare the infectivities of MYXV-Lau and MYXV-Tol and to examine whether *M159* gene expression also has an effect on the replication and spread of MYXV in rabbit cells, several recombinant viruses were evaluated in two different rabbit cell lines: RK13, a rabbit kidney-derived cell line, and RL-5, a rabbit CD4^+^ T cell line. These two rabbit cell lines were infected with the six types of recombinant viruses described above (Table 1) at an MOI of 1 (one step) or an MOI of 0.1 (multistep) (Fig. S5). In contrast to what was observed in hare PBMCs and HN-R cells (Fig. 3A and B), all tested viruses/viral constructs replicated efficiently and to similar levels in the permissive rabbit RK13 cell line, regardless of the MOI used (Fig. S5A and B). A similar scenario was observed for the rabbit RL-5 cell line, where all viruses exhibited identical transmission kinetics and reached similar titers at 72 hpi (Fig. S5C and D). These results suggest that in the rabbit cell lines tested, neither the expression of the *M159* gene nor the different MYXV backbones have detectable effects on the overall replication efficiency of these recombinant viruses in rabbit cells.

10.1128/mbio.03461-21.5 FIG S5 M159 does not alter MYXV infection in rabbit RK13 and RL-5 cells. Single- and multiple-step growth curves of MYXV infection in RK13 and RL-5 T lymphocytes are shown. The indicated cells were infected with vMyxTol, vMyxTol-Δ159, vMyxLau, vMyxLau-V5M159, vMyxLau-ΔM009+recTol, and vMyxLau-recTol at MOIs of 1 and 0.1 and then collected at 0, 8, 24, and 48 hpi. The virus titers are representative of results from three independent experiments and were determined in triplicate following serial dilution onto RK13 cells. Significant differences among means were determined using two-way ANOVA followed by Dunnett’s test using Prism 6 (GraphPad Software, Inc.). Differences at a *P* value of <0.05 were considered significant. Download FIG S5, PDF file, 0.5 MB. Copyright © 2022 Águeda-Pinto et al. 2022 Águeda-Pinto et al. https://creativecommons.org/licenses/by/4.0/ This content is distributed under the terms of the Creative Commons Attribution 4.0 International license.

Since the 1950s, MYXV has evolved rapidly and converged to less pathogenic phenotypes in the feral European rabbit populations in both Australia and Europe (28). More recently, Alves et al. (29) also showed that the resistance of European rabbit populations to MYXV evolved by a strong pattern of parallel evolution, with selection on immunity-related genes favoring the same alleles in Australia, France, and the United Kingdom. As mentioned above, apart from the inserted recombinant region of unknown origin, the MYXV-Tol backbone is ∼99% similar to the MYXV-Lau strain, and therefore, it is possible that the European rabbit populations in Spain and Portugal have undergone long-term adaptation to MYXV strains. Moreover, our results show that M159 does not contribute to MYXV host range functions in the tested rabbit cells, which might indicate that in the native environment, there is no difference in the pathogenicity of MYXV-Tol compared to those of other MYXV strains in European rabbit populations. Nevertheless, future *in vivo* experimentation in rabbits will need to be conducted to understand the clinical outcome of myxomatosis for the different MYXV constructs.

### M159 upregulates the replication of MYXV-Lau and MYXV-Tol in selected human cancer cells.

**FIG 6:**
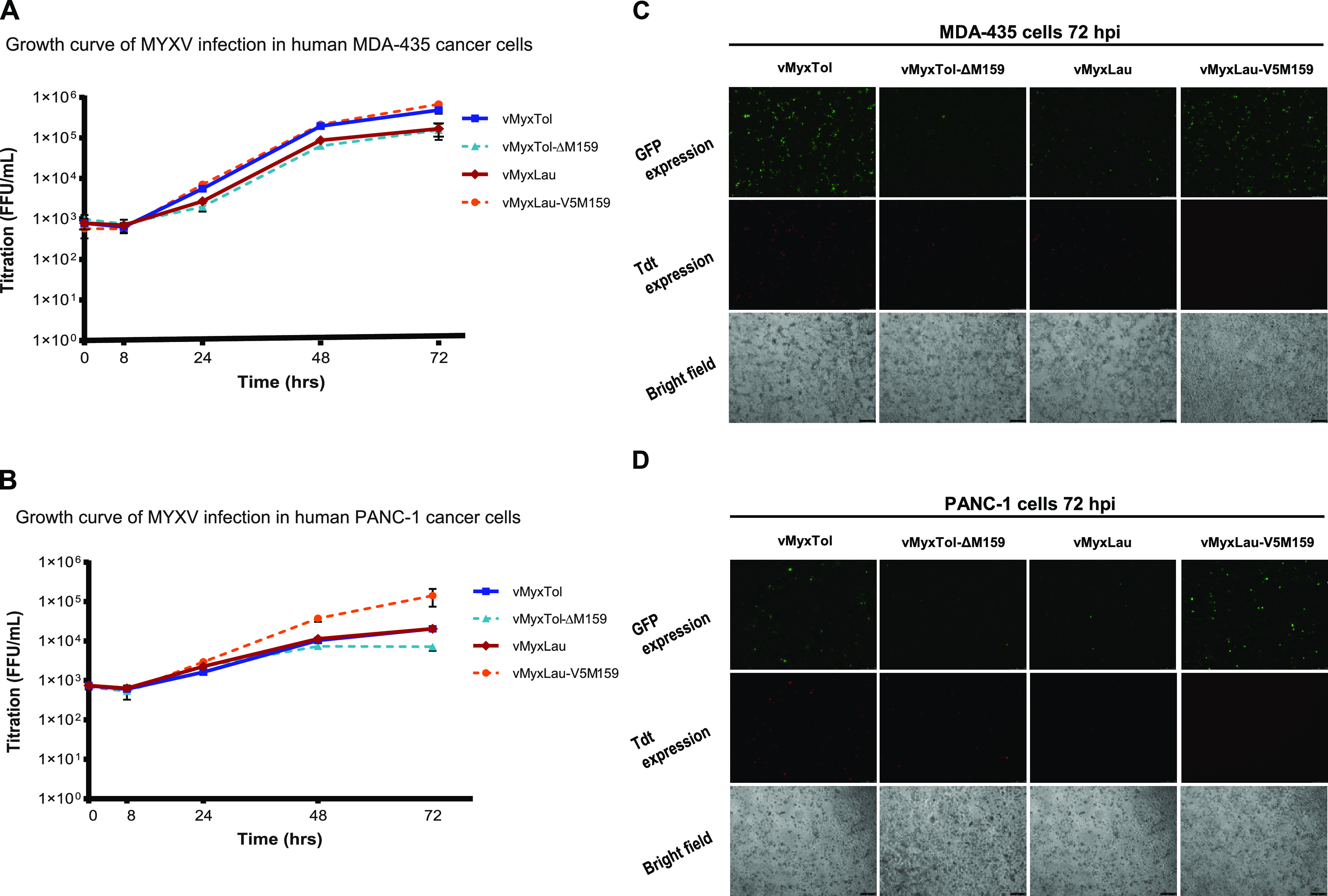
M159 alters the replication of MYXV in human cancer cells. (A and B) Single-step growth curves of MYXV in MDA-435 (A) and PANC-1 (B) human cancer cell lines. The cultures were infected with vMyxTol, vMyxTol-Δ159, vMyxLau, and vMyxLau-V5M159 at an MOI of 1, and the cells were then collected at 1, 8, 24, 48, and 72 hpi. Virus titers are representative of results from three independent experiments and were determined in triplicate following serial dilutions onto RK13 cells. Significant differences among means for the experiments in panels A and B were determined using two-way ANOVA followed by Dunnett’s test using Prism 6 (GraphPad Software, Inc.). Differences at a *P* value of <0.05 were considered significant. (C) Fluorescence microscopy images of infected MDA-435 cancer cells. The cells were infected with vMyxTol, vMyxTol-Δ159, vMyxLau, and vMyxLau-V5M159 at an MOI of 1, and the images were taken using an inverted fluorescence microscope at a ×5 magnification at 72 hpi. (D) Fluorescence microscopy images of infected PANC-1 cancer cells. The cells were infected with vMyxTol, vMyxTol-Δ159, vMyxLau, and vMyxLau-V5M159 at an MOI of 1, and the images were taken using an inverted fluorescence microscope at a ×5 magnification at 72 hpi.

Despite MYXV being able to infect only leporids in nature, MYXV can selectively enter and kill cancer cells from different nonrabbit species, including mice and humans (30). For this reason, different multiple-transgene-armed recombinant MYXV constructs are currently being developed as oncolytic therapeutics for human cancer (31). Based on our observations that the *M159* gene plays a major role in the ability of MYXV to replicate in hare cells (Fig. 3), we tested the infection and replication of different recombinant MYXVs (Table 1) in a variety of human cancer cell lines that are considered to be nonpermissive or semipermissive to the parental MYXV-Lau strain (Fig. 6). The parental virus (vMyxLau) replicates poorly in the human pancreatic cancer cell line PANC-1 and the human melanoma cancer cell line MDA-435 (32). As shown in Fig. 6, the presence of *M159* in the MYXV-Lau backbone significantly increased MYXV yields in both the PANC-1 and MDA435 cell lines after 48 and 72 hpi, suggesting that the expression of M159 can, to some extent, confer replication competence of MYXV-Lau in these nonpermissive human cell lines. Interestingly, cell viability was unchanged in both cell lines when infected with the different recombinant viruses (data not shown). When the MDA-435 cell line was infected with wild-type vMyxTol, higher transmission kinetics and higher progeny virus titers than with vMyxLau infection were also observed (Fig. 6A and C). Interestingly, in PANC-1 cancer cells, infection by vMyxTol reached transmission kinetics similar to those of vMyxLau (Fig. 6B). The fact that *Serp-3* is disrupted in the MYXV-Tol backbone might partially explain the similar titers obtained for infection with vMyxLau (7). However, it is also possible that other mutations in the MYXV-Tol backbone might decrease the overall infectivity of this virus in this human cell line, even with the expression of the *M159* gene. As MYXV is being developed as an oncolytic therapeutic for different human cancers, the discovery that M159 can increase MYXV replication in selected semipermissive or nonpermissive human cancer cell lines can open new doors for future studies on the potential use of MYXV as an oncolytic agent. Moreover, given that M159 exhibits host range properties in both hare and human cells, it is possible that the host target pathway of M159 might be conserved across species, which will allow us to continue our efforts in the identification of M159-targeted pathways in a model that is easier to study and experiment with.

### Concluding remarks.

The presence of novel C7L host range gene family members in an MYXV strain (Toledo) capable of crossing the species barrier and causing myxomatosis in Lepus granatensis raises some relevant questions regarding the host range abilities of this new virus. In this study, we have shown that the ability of MYXV-Tol to infect hare cells (and, to a lesser extent, normally nonpermissive human PANC-1 and MDA-435 cancer cells) and replicate ultimately depends on the M159 protein alone. Interestingly, the M159 protein did not contribute to increased replication in the tested permissive rabbit cells. Our studies with wild-type MYXV-Lau or vMyxTol-ΔM159 showed that both viruses were unable to detectably replicate in hare cells, while MYXV-Tol that expresses M159 underwent fully productive replication. Interestingly, in primary hare PBMCs, the insertion of the *M159* gene into the MYXV-Lau backbone was not enough to rescue MYXV-Lau infection in these cells, raising awareness of the need for additional replication requirements in this hare cell type. From the results shown here, M159 expression was not required for MYXV binding, entry, or early gene expression, but it was necessary for relieving an early block of viral replication and late protein synthesis in hare HN-R cells.

Our three-dimensional (3D) modeling analyses show that the predicted M159 protein has the common protein scaffold that characterizes other C7L family members but presents amino acid differences in its sequence at the three-fingered molecular claw, which is responsible for the ability of some C7L family members to bind to the host restriction factor SAMD9. Moreover, while the results show that *M159* is an early/late gene, the M159 protein can accumulate at high levels only at later time points. Considering the migration of M159 to the nucleus from the cytoplasmic compartment, it is possible that, besides its ability to bind the hare version of SAMD9, M159 might also bind or induce a cellular factor(s) that mediates host range or immunosuppression at later time points from the nuclear compartment of hare cells. M159 was derived from an unknown donor poxvirus that in terms of gene cassette order resembles members from the ungulate poxvirus family. For this reason, it is possible that in its “natural” host, the M159 protein acts by regulating a unique repertoire of antiviral response pathways that confer advantages in engaging and modulating the hare’s antiviral responses. Nevertheless, although we have been unable to demonstrate a stable protein-protein interaction complex between M159 and SAMD9 in hare cells, a putative transitory interaction with the hare version of SAMD9 cannot be discarded.

Several relevant technical and logistical limitations exist when working with a hare model: first, it is very difficult to establish hare colonies to test MYXV pathogenicity, and second, the absence of an available hare genome in online databases and the lack of reagents optimized for hare cell lines make it very difficult to study possible M159 interactions with the host pathway. In the future, the use of human cell lines might provide a valuable resource for the study of the mechanism of M159 action in host cells. Nevertheless, our results provide strong evidence that the M159 protein found in the recombinant region of MYXV-Tol is the prime candidate enabling this recombinant MYXV-Tol to infect hare cells, which ultimately might culminate in myxomatosis in Iberian hares. We believe that these data have the potential to improve the current knowledge about the host range and virulence of poxviruses in general and provide a platform for better understanding the pathogenicity and transmission success of emerging viruses like the new MYXV-Tol virus, rendering it capable of leaping into a new host species. Moreover, the presence of this novel host range factor in an MYXV strain capable of crossing the species barrier suggests that the acquisition of this gene had a preponderant role in this species leap and highlights that homologous recombination could also be a feature of gene gain that might play a major role in poxvirus evolution.

## MATERIALS AND METHODS

### Phylogenetic analysis and homology modeling of C7-like proteins of MXYV.

The M159 amino acid sequence and all other representative related protein sequences (*n* = 34) encoded by poxviruses were downloaded and aligned using MAFFT. The resulting alignment was used to infer a maximum likelihood phylogenetic tree using PHYML (33) with JTT+G+F (Jones–Taylor–Thornton model, where +G stands for using gamma rate heterogeneity and +F for using observed frequencies) used as the amino acid substitution model, determined by ProtTest (34). Branches with <0.8 approximate likelihood ratio test (aLRT) branch support were collapsed using TreeGraph2 (35).

The amino acid sequences of C7L proteins (M159, M64, M63, and M62) were used for modeling with the structures under Protein Data Bank accession no. 5CZ3B (crystal structure of myxoma virus M64) and 5CYWB (crystal structure of vaccinia virus C7 protein) using Phyre2 (36). The structures were aligned and visualized with the PyMOL molecular graphics system v2.4 (Schrödinger, LLC).

### Construction of recombinant viruses.

Based on our findings that the MYXV-Tol variant contains a new genomic cassette insertion, including a tentative candidate host range factor (M159), we constructed several MYXV recombinants to assess whether the M159 protein influences MYXV-Tol infectivity in cells from different leporids. For this, we constructed two different sets of recombinant viruses: viruses with MYXV-Lau as the backbone and viruses based on MYXV-Tol as the backbone (7) (Table 1).

In the recombinant virus vMyxTol-ΔrecTol-GFP, the entire 2.7-kb recombinant cassette (recTol) from MYXV-Tol was removed and replaced with the GFP gene under the control of a poxvirus p11 late promoter. In wild-type MYXV-Tol, a reporter cassette that expresses TdT (37) under the control of a poxvirus late p11 promoter and the GFP gene under the control of a poxvirus early-late promoter was inserted between the *M135R* and *M136R* genes (vMyxTol-TdT-GFP). The same reporter cassette was also inserted into the *M159* locus of MYXV-Tol, which resulted in an *M159* gene knockout recombinant MYXV-Tol (vMyxTol-ΔM159-TdT-GFP). In the MYXV-Lau backbone, the new recTol cassette was inserted into the *M009L* locus (vMyxLau-ΔM009-TdT-recTol-GFP) along with the TdT gene (late p11 promoter) and the GFP gene (early-late promoter), which disrupts *M009L* and allows the expression of all the viral proteins from the novel 2.8-kb recombinant cassette derived from MYXV-Tol. In another construct, which acted as a control, the full recTol cassette was inserted between the *M135* and *M136* gene loci of MYXV-Lau (vMyxLau-recTol-TdT-GFP). To characterize M159 expression in hare cells, recombinant MYXVs were constructed expressing the V5-tagged *M159* gene under the control of its own promoter and the GFP gene under the control of an early-late promoter, which were inserted between the *M135L* and *M136L* gene loci of both MYXV backbones (vMyxLau-V5tagM159-GFP and vMyxTol-V5tagM159-GFP). Wild-type MYXV-Lau expressing TdT (late p11 promoter) and GFP (early-late promoter) between the *M135L* and *M136L* loci (vMyxLau-TdT-GFP) was used for comparison (38).

For all recombinant viruses, recombinant plasmids were first constructed using the MultiSite Gateway Pro system (Invitrogen), as described previously (27, 39, 40), or the Gibson cloning method (New England BioLabs, USA). Upstream and downstream hybridizing sequences were amplified by PCR using specific primers to create entry clones by the Gateway BP recombination system with appropriate pDONR vectors. The final recombination plasmids were obtained by recombination of the three pDONR entry vectors with a destination vector (pDEST40; Invitrogen), using the Gateway LR recombination system. Subconfluent monolayers of RK13 cells were then infected with the appropriate MYXV backbone and transfected with the final recombination plasmids using Effectene transfection reagent (Qiagen, USA) or Lipofectamine 2000 (Thermo Fisher, USA) according to the manufacturer’s instructions. Confirmation of a successful recombinant virus clone was achieved by PCR amplification, Sanger sequencing, and subsequent focus purification. Purification of the recombinant virus stocks was carried out using 36% (wt/vol) sucrose cushions. All recombinant viruses showed fully permissive replication in the RK13 cell line. The viral titers were quantified by counting foci (focus-forming units [FFU] per milliliter), areas of virus-infected cells, under a fluorescence microscope.

### Cell lines and cell culture.

Rabbit cells (cell line RK13; ATCC CCL-37) and MDA-435 (cell line MDA-MB-435; ATCC HTB-129) and PANC-1 (32) human cancer cells were cultured in Dulbecco minimum essential medium (DMEM; Invitrogen, USA) supplemented with 10% fetal bovine serum (FBS; Gibco, USA), 2 mM glutamine (Invitrogen, USA), and 100 μg/mL of penicillin-streptomycin (Pen/Strep; Invitrogen, USA). Hare cells (cell line HN-R; Friedrich Loeffler Institut, Germany), a spontaneously immortalized cell line derived from kidney (41), were cultured in 50% Iscove’s modified Dulbecco’s medium (IMDM) and 50% Ham’s F-12 medium supplemented with 10% FBS, 2 mM glutamine, and 100 μg of Pen/Strep per mL. All cultures were maintained at 37°C in a humidified 5% CO_2_ incubator (Thermo Fisher, USA). The RL-5 rabbit CD4^+^ T cell line was cultured in RPMI 1640 (obtained from Gibco), also supplemented with 10% FBS, 2 mM glutamine, and 100 g/mL of Pen/Strep.

### Isolation of hare PBMCs and infection with viruses.

Blood samples were obtained during the sanitary management of Iberian hare individuals from approved breeding hares. Blood was collected from healthy unvaccinated (namely, juveniles) and vaccinated (adults) Iberian hares. Animals were caught with hare-specific nets and handled under dark conditions to reduce stress. Blood was collected by puncture from the marginal vein of the ear and immediately transferred to sodium citrate vacutainers or mixed with an equal volume of 6.4% anticoagulant sodium citrate. The blood sample was diluted with the same volume of sterile 1× phosphate-buffered saline (PBS) with 5% FBS and mixed thoroughly. Next, diluted blood was carefully layered in sterile falcon tubes with Lymphoprep and centrifuged at 1,200 × *g* for 10 min at 20°C, with the brakes turned off. Layers corresponding to plasma cells, mononuclear cells, granulocytes, and Lymphoprep were transferred to a sterile centrifuge tube, mixed with the same volume of 1×PBS with 5% FBS, and centrifuged at 400 × *g* for 10 min at 25°C. Virus infections were performed in complete RPMI 1640 culture medium. For virus infections, PBMCs were incubated with the test recombinant MYXVs at a multiplicity of infection (MOI) of 10 at 37°C for 1 h to allow virus adsorption and washed twice with complete RPMI 1640 to remove unbound virus, and cells were then resuspended in the desired volume of medium and incubated at 37°C for virus infection. Cells were monitored under a fluorescence microscope or used for virus titration. Virus titrations of the samples from hare PBMCs were performed using RK13 cells, where all the test viruses demonstrated permissive replication.

### One-step and multiple-step viral growth curves.

One-step growth curves were made by quantifying viral yields at various time points from HN-R or RK13 cells infected with virus at various MOIs. After 1 h of incubation with the virus inoculum, the cells were washed with PBS, and the medium was replaced with the appropriate growth medium. Cells were then collected at the desired time points. Multiple-step growth curves were made by titrating virus yields from cell lysates harvested at various time points after infection at an MOI of 0.1. Titrations were conducted using HN-R or RK13 cells, as described previously (14). Cytosine arabinoside (AraC; Sigma Life Science) was used to pretreat cells (40 μg/mL) for 1 h before viral infection.

### Microscopy.

To process the hare HN-R cells for immunofluorescence microscopy, cells were washed twice with PBS and fixed with 2% formaldehyde, prepared fresh in PBS from paraformaldehyde, for 12 min at room temperature (RT). The cells were then permeabilized for 1 min at RT in PBS containing 0.1% Triton X-100, washed twice with PBS, and blocked with 3% bovine serum albumin (BSA) in PBS for 30 min at 37°C. The cells were then incubated with V5 primary antibody (Invitrogen, USA) at 4°C overnight, washed six times with PBS, and incubated with Alexa Fluor 633 anti-goat secondary antibody (Thermo Fisher, USA) for 30 min at 37°C. Nuclei and viral factories were then stained with 4′,6-diamidino-2-phenylindole (DAPI), and the coverslips were sealed with nail polish. Samples were imaged with a Nikon C2 scanning confocal or Nikon Ti microscope, using a 60× Plan Apo water immersion lens with a numerical aperture of 1.2. Excitation lasers of 405 nm, 488 nm, and 640 nm paired with blue, green, and far-red detectors were used to detect DAPI, GFP, and immunolabeled V5-tagged M159, respectively. Images were postprocessed using Nikon Elements software.

### Western blot analysis.

The mock and infected cells were harvested at different time points after infection with viruses, washed with PBS, and stored at −80°C or processed immediately with radioimmunoprecipitation assay (RIPA) lysis buffer (50 mM Tris, 150 mM NaCl, 0.1% SDS, 0.5% sodium deoxycholate, 1% NP-40, 1 mM phenylmethylsulfonyl fluoride [PMSF], and a protease inhibitor cocktail [Roche, USA]). The concentration of total proteins was estimated by a Bradford assay (Bio-Rad, USA), and equal amounts of total protein were used for Western blot analysis as described previously (42). Commercial antibodies targeting anti-V5 (Invitrogen, USA), antiactin (Ambion, USA), goat anti-mouse IgG conjugated with peroxidase (Thermo Scientific, USA), and goat anti-rabbit IgG conjugated with peroxidase (Santa Cruz Biotechnology, USA) were used for this study. Antibodies against MYXV SERP-1 and MT-7 were previously described (14). Samples were analyzed on 10% SDS-PAGE gels and transferred to a polyvinylidene difluoride (PVDF) membrane (GE Healthcare, USA) using a wet-transfer apparatus (Invitrogen, USA). The membranes were blocked with 5% nonfat milk in Tris-buffered saline (TBS) buffer (20 mM Tris, 150 mM NaCl [pH 7.6]) containing 0.1% Tween 20 (TBST) (Fisher Scientific, USA) for 1 h at room temperature. Following this, membranes were incubated at 4°C overnight with the primary antibody at the appropriate dilution, washed three times with TBST, and incubated with the secondary antibody conjugated to horseradish peroxidase (HRP) for 1 h at room temperature. After washing, proteins of interest were detected using the Immobilon Western HRP substrate (Millipore) on Kodak BioMax film (Carestream Health, Inc.).

### DNA and RNA extraction and quantitative PCR.

To quantify viral loads and gene expression, nucleic acid was extracted from RK13 and HN-R cell lines using the Qiagen DNeasy blood and tissue kit (Qiagen, USA). Quantitative real-time PCR was set up using SsoAdvanced universal SYBR green supermix (Bio-Rad, USA) targeting a region in the M53 myxoma virus gene (M53F [5′-CTATCGCGTCGTTAAACAAATTGC-3′] and M53R [5′-GTTGTCGTCCTTATCCATGATAATTCG-3′]) that is conserved in MYXV-Tol and -Lau. Each reaction mixture contained 10 μL of SsoAdvanced universal SYBR green supermix (Bio-Rad, USA), 0.4 μL (10 μM) of forward and reverse primers, 5.2 μL of water, and 4 μL of DNA. The pJET 1.2 vector containing the target M53 region was used as a standard. The assay mixture was run on a Bio-Rad CFX96 real-time thermal cycler under cycling conditions according to the SsoAdvanced universal SYBR green supermix manufacturer’s recommendations, with a melt curve included.

RNA was extracted from infected HN-R cells using a Qiagen RNeasy minikit (Qiagen, USA). Reverse transcription-qPCR (RT-qPCR) for assessing the gene expression of *M159* and *Serp-1* in vMyxTol-infected HN-R cells was undertaken using SuperScript III first-strand synthesis supermix (Thermo Fisher Scientific, USA). Primers and probes targeting the *M159* gene region (M159F [5′-GGTTTTACTTTTACCACTTCACTCC-3′] M159R [5′-GAGGGGACAGTTATGGATGTAC], and M159Probe [5′-FAM {6-carboxyfluorescein}-CCGGTTCCAACACAATAACG-3′-BHQ1 {black hole quencher 1}]) and the *Serp-1* gene region (Serp-1F [5′-CAATAGCAGCATTTTAGTATCTCGGTCTAG-3′], Serp-1R [5′-GTCATTAACTCGTACGTTAAGGATAAGACG-3′], and Serp-1Probe [5′-FAM-GTCCAATACGCGTGGGACGTCTC-3′-BHQ1]) were used in separate assays. The assay mixtures consisted of the following master mix components per reaction: 12.5 μL of 2× RT reaction mix, 0.5 μL of SuperScript III first-strand synthesis supermix (Thermo Fisher Scientific, USA), 0.5 μL (10 μM) of forward and reverse primers, 0.25 μL (10 μM) of the probe, 6.75 μL of water, and 4 μL of RNA. Standards for both target regions, M159 and Serp-1, were generated using the pJET 1.2 vector. qPCR was performed on a Bio-Rad CFX96 real-time thermal cycler under the cycling conditions recommended by Thermo Fisher Scientific for the SuperScript III first-strand synthesis supermix.
