# Supplementary figures and images for: Identification of a Novel Myxoma Virus C7-Like Host Range Factor That Enabled a Species Leap from Rabbits to Hares
Source: mBio. 2022 Mar 30;13(2):e03461-21. doi: 10.1128/mbio.03461-21 (PMC9040879; doi:10.1128/mbio.03461-21)

**A**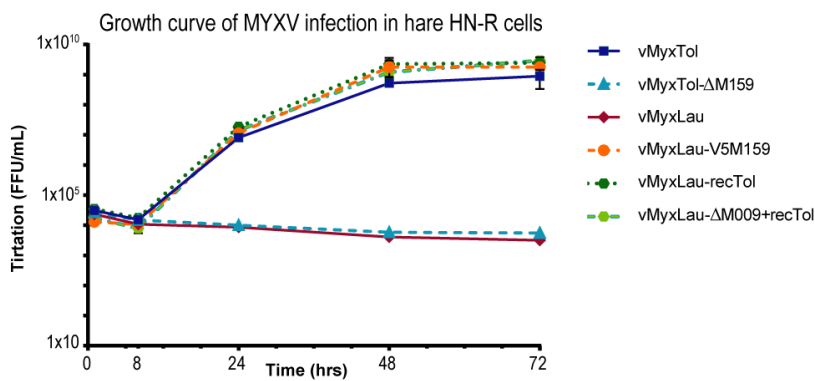**B**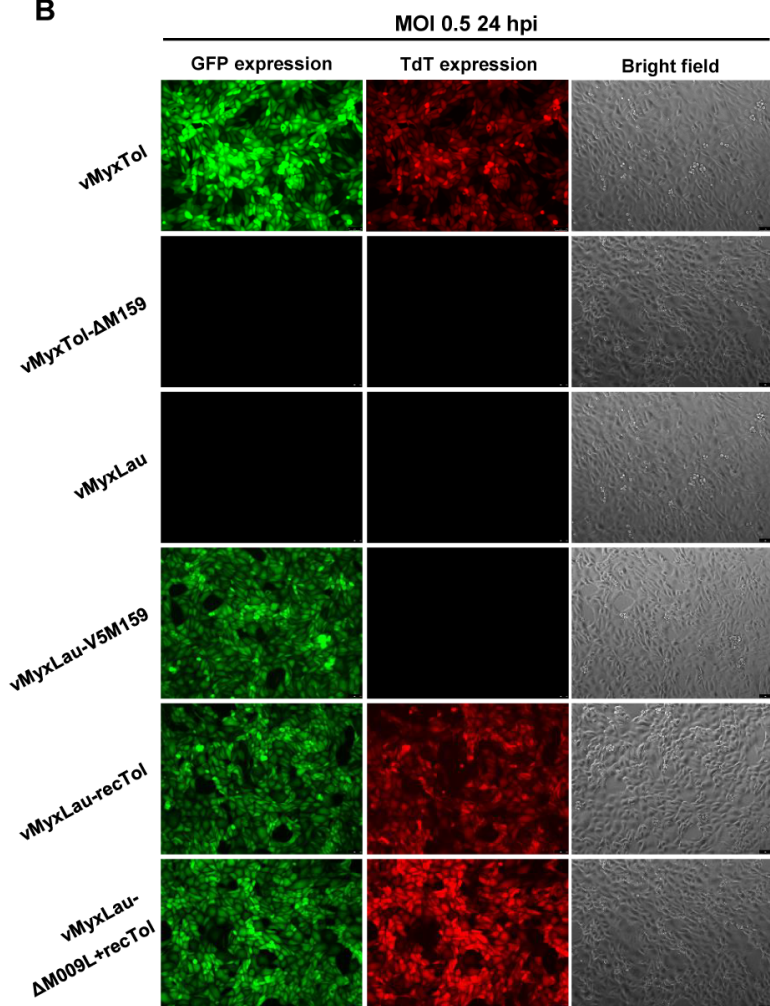

Supplement: FIG S1 [file mbio.03461-21-sf001.pdf]

MOI 5 24 hpi

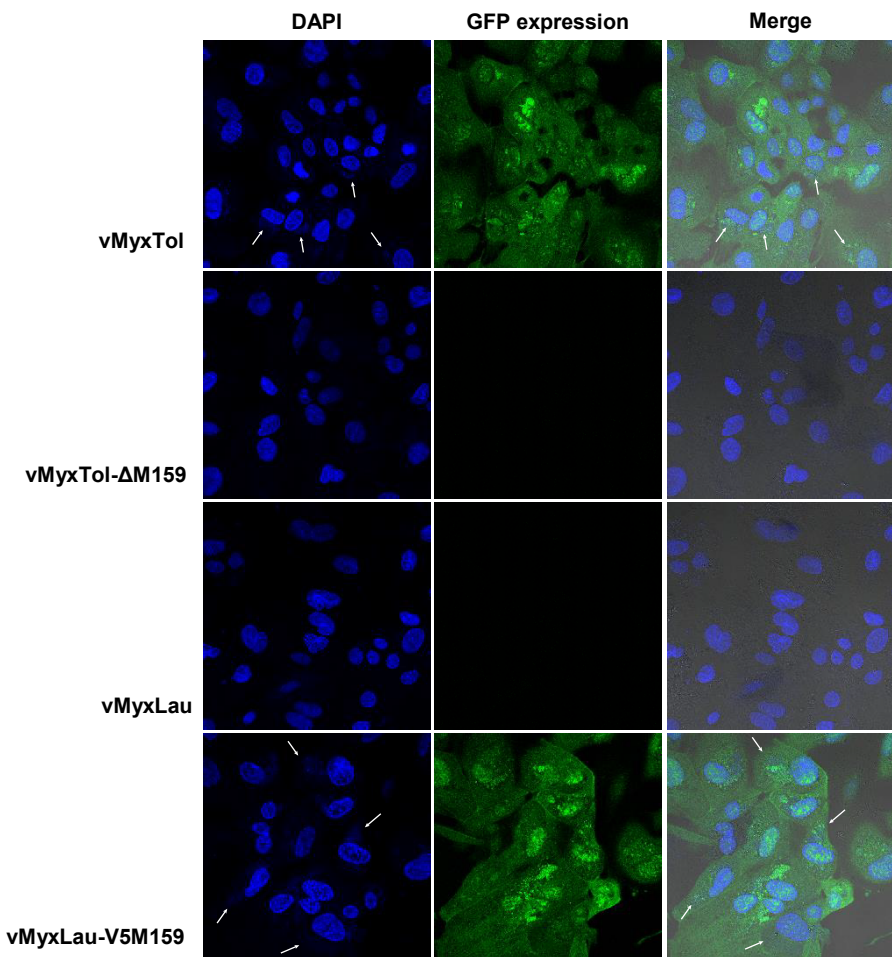

Supplement: FIG S2 [file mbio.03461-21-sf002.pdf]

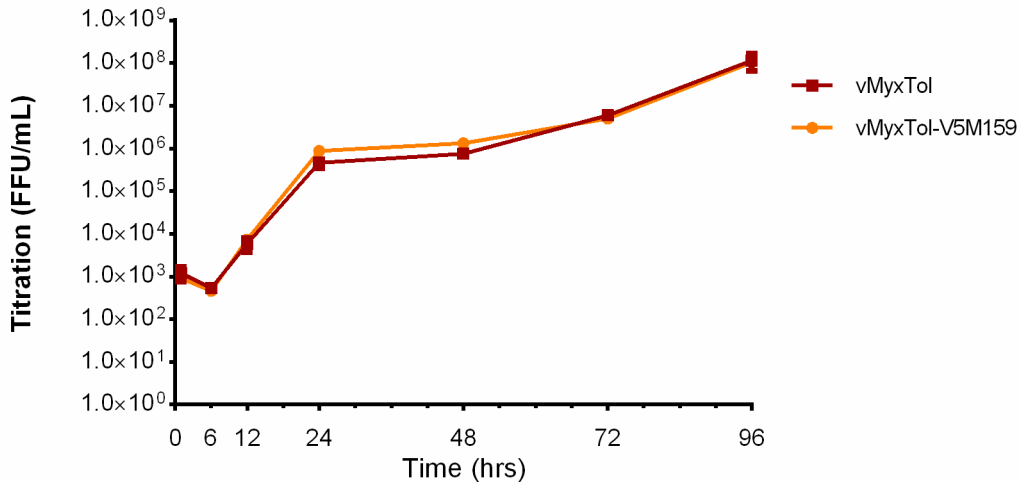

Supplement: FIG S3 [file mbio.03461-21-sf003.pdf]

**vMyxTol-V5M159**

**+AraC**

1hr

3hr

8hr

24hr

48hr

24hr

48hr

## V5 tagged M159

## Actin

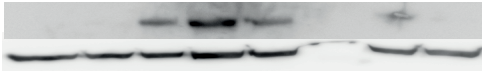

Supplement: FIG S4 [file mbio.03461-21-sf004.pdf]
