# Supplementary material for: Identification of a Novel Myxoma Virus C7-Like Host Range Factor That Enabled a Species Leap from Rabbits to Hares
Source: mBio. 2022 Mar 30;13(2):e03461-21. doi: 10.1128/mbio.03461-21 (PMC9040879; doi:10.1128/mbio.03461-21)
Supplement: FIG S5 [file mbio.03461-21-sf005.pdf]

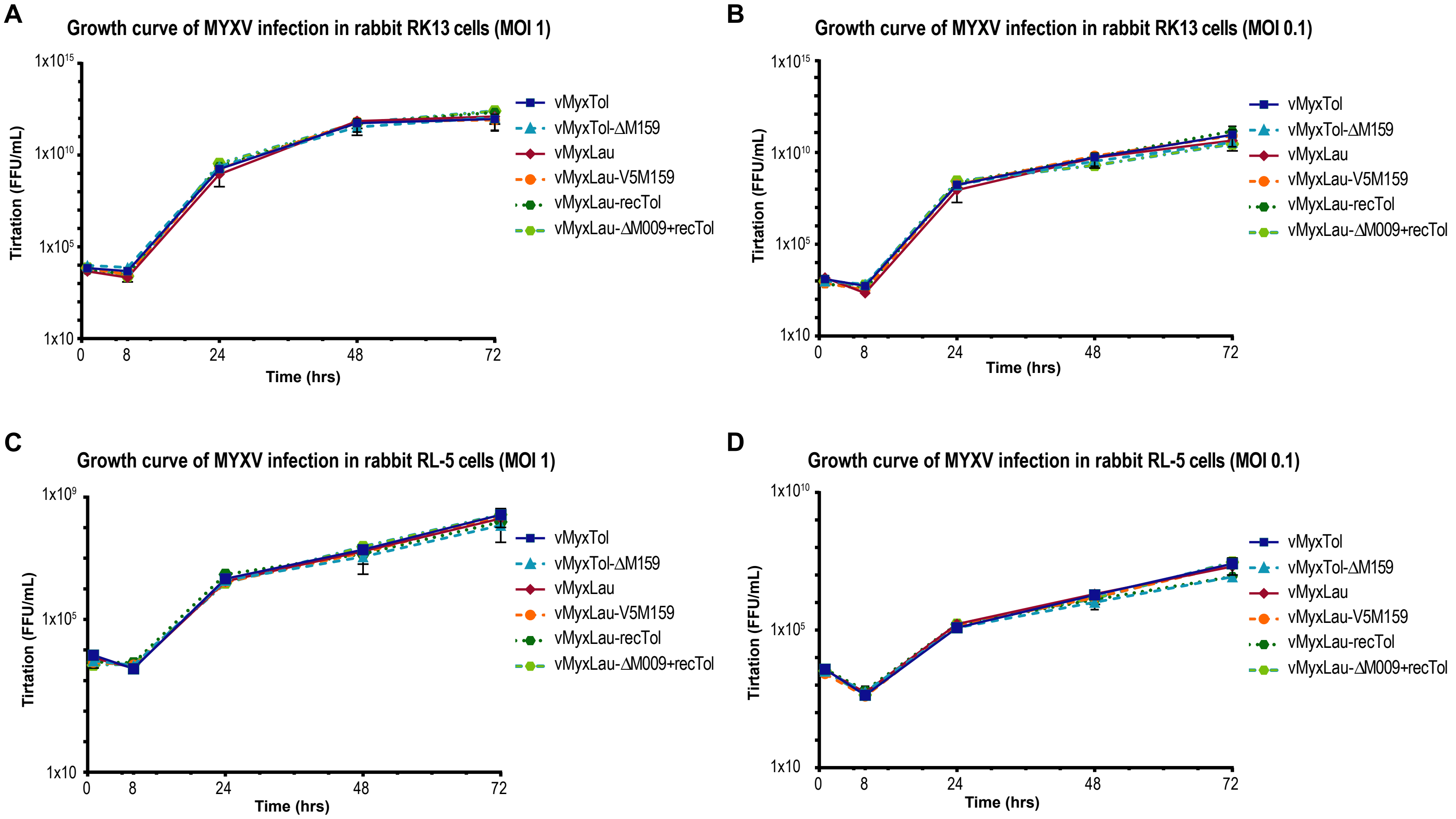
A
Growth curve of MYXV infection in rabbit RK13 cells (MOI 1)
B
Growth curve of MYXV infection in rabbit RK13 cells (MOI 0.1)
C
Growth curve of MYXV infection in rabbit RL-5 cells (MOI 1)
D
Growth curve of MYXV infection in rabbit RL-5 cells (MOI 0.1)
Tirtation (FFU/mL)
Time (hrs)
0
8
24
48
72
1x10
1x10^5
1x10^9
1x10^10
1x10^15
vMyxTol
vMyxTol-ΔM159
vMyxLau
vMyxLau-V5M159
vMyxLau-recTol
vMyxLau-ΔM009+recTol
